# Pediatric retinal detachment in the Eastern Province of Saudi Arabia: experience of a tertiary care hospital

**DOI:** 10.4103/0256-4947.55165

**Published:** 2009

**Authors:** Rizwan A. Cheema, Wajeeha Al-Khars, Essam Al-Askar, Yasir M. Amin

**Affiliations:** From the Dhahran Eye Specialist Hospital, Dhahran, Saudi Arabia

## Abstract

**BACKGROUND AND OBJECTIVES:**

Because no previous studies have addressed the issue, we describe clinical characteristics and surgical outcome of patients with rhegmatogenous retinal detachment (RRD) in a pediatric population of the Eastern province of Saudi Arabia.

**PATIENTS AND METHODS:**

We conducted a retrospective review of all consecutive cases of pediatric RRD (0-18 years) patients presenting at Dhahran Eye Specialist Hospital, a tertiary care hospital, in the Eastern Province of Saudi Arabia over a period of 3 years.

**RESULTS:**

Twenty patients were included in the study, accounting for 9.4% of all retinal detachment surgery cases performed over a period of 3 years (January 2006 to December 2008). The median age was 11.0 years, (range, birth to 18 years). Trauma, (45%) myopia/vitreoretinal degeneration (10%) and prior ocular surgery (25%) were significant risk factors for RRD. Proliferative vitreoretinopathy (PVR) more than grade C was present in 14/20 (70%) of cases. Most patients (15/20, 75%) were treated with pars plana vitrectomy and placement of an encircling buckle, while silicone oil or gas was used as tamponade in 13/20 (65%) patients. Surgery was successful in 17/20 (85%) cases in achieving retinal re-attachment. Visual acuity improved significantly following surgery (Mean preop 2.146 LogMAR, Mean postop 1.497 LogMAR) (*P*=.014). Longer duration of RRD (*P*=.007) and macular involvement (*P*=.05) were associated with worse anatomical outcomes following surgery.

**CONCLUSION:**

Pediatric RRD in the Eastern province is often associated with predisposing pathology. Surgery is successful in achieving anatomical reattachment of the retina in a majority of cases with improvement of visual acuity.

Pediatric rhegmatogenous retinal detachment (RRD) accounts for 2.0% to 6.6% of all RRD cases.^1^–^3^ In contrast to RRD in adults, children usually present late and have clinical features attributable to a longstanding retinal detachment such as macular involvement and proliferative vitreoretinopathy (PVR).^4^ Consequently, guidelines obtained from studies of adult cases may be inappropriate for applying to management of pediatric RRD. No previous studies describing clinical features and surgical outcome of pediatric patients with RRD have been reported in Arabian patients. We report clinical features and surgical outcome of pediatric RRD on a consecutive series of patients undergoing retinal redetachment surgery in the eastern province of Saudi Arabia.

## PATIENTS AND METHODS

We conducted a retrospective chart review of all consecutive pediatric patients (younger than 18 years of age) who had surgery for retinal detachment at the Dhahran Eye Specialist Hospital, a tertiary care hospital in the eastern province of Saudi Arabia, during a period of 36 months from January 2006 to December 2008. Patients in the study were identified by an operating room log, and all operations were performed by a single experienced vitreoretinal surgeon (RAC).

A total of 20 patients were included in the study. Clinical records of patients were reviewed and information regarding age, sex, preoperative visual acuity (Snellen converted to LogMAR), characteristics of retinal detachment, retinal tears, surgical procedures, tamponade, lens status, predisposing ocular conditions, PVR and prior intraocular surgery, final visual acuity and retinal reattachment status of last patient visit was recorded. Four patients with less than three months of postoperative follow-up were excluded from the study.

Statistical analysis of results was done with SPSS statistical software (version 13.0.1, Professional Statistics Release, Chicago, USA). The Wilcoxen signed ranks test was used for comparison of means for numerical data and the Fisher exact test for categorical data. A significance level of .05 was used in this study.

## RESULTS

A total of 20 patients were included in the study (14 males, 6 females), and they accounted for 9.4% of all retinal detachment surgery cases performed over a period of 3 years. The duration of postoperative follow-up ranged from 6 to 36 months (mean, 24.4 months). Table 1 shows the demographics of the patients. Table 2 shows clinical features of retinal detachment at presentation. The retinal detachment was total or subtotal at presentation in most patients (17/20), with macular involvement in over half of the patients. The duration of retinal detachment was unknown in 12/20 (60%) of patients, while 4/10 (20%) patients had retinal detachment for less than two months duration and 4/20 (20%) had retinal detachement for than two months. PVR more than grade C was present in 14/20 (70%) of cases. The main predisposing factors associated with retinal detachment included blunt trauma (9/20, 45%), prior intraocular surgery (5/20, 25%) and myopia/vitreoretinal degeneration (4/20, 20%). Two cases (2/20, 10%) were not associated with any identifiable predisposing condition, while retinopathy of prematurity (1/20, 5%) and Stickler syndrome (1/20, 5%) were responsible for others. Table 3 shows type of surgical procedure performed. Most patients (15/20, 75%) were treated with internal approach with pars plana vitrectomy in association with placement of encircling buckle, while silicone oil or gas used as tamonade in 13/20 (65%) patients. Successful retinal reattachment was obtained in 17/20 (85%) cases, which was reflected in improvement of mean visual acuity (*P*=.018) from preoperative levels.

**Table 1:** Preoperative characteristics.

Number of patients	20
Age	Mean=8.68 years; SD=7.007; Median=11.0 years
	(Range: 0.1-18.0)
Sex	
Male	14 (70%)
Female	6 (30%)
Preoperative visual acuity	Mean=2.146 LogMAR
	(Range=0-3.0)
Lens status	
Phakic	15 (75%)
Aphakic	4 (20%)
Pseudophakic	1 (5%)

**Table 2:** Clinical characteristics of retinal detachment.

Number of retinal breaks	
>2 tears	8 (40%)
Not seen	6 (30%)
One tear	6 (30%)
Type of retinal breaks	
Retinal tear	13 (65%)
Retinal hole	3 (15%)
Giant retinal tear	3 (15%)
Retinal dialysis	1 (5%)
PVR (>Grade C)	
Present	14 (70%)
None	6 (30%)

**Table 3:** Surgical procedure.

Type of surgical procedure	
PPV+SB	15 (75%)
Scleral buckle (SB)	4 (20%)
Pars plana vitrectomy (PPV)	1 (5%)
Tamponade used	
Silicone oil	9 (45%)
None	7 (35%)
Gas. (C3F8)	4 (20%)

Table 4 shows factors associated with worse anatomical outcome. Two factors were associated with worse anatomical outcomes. These included macular detachment (*P*=.05) and longer duration of retinal detachment (*P*=.007) at presentation. Although not statistically significant, a trend of worse anatomical outcome was also seen in patients in whom retinal breaks were not seen or more than two retinal breaks were present. (*P*=.089)

**Table 4:** Factors associated with worse anatomical outcome.

Risk factors	Retina attached		
	Yes	No	*P* value
All patients	17	3	
Preoperative characteristics			
Age (Mean years, range)	9.25 (0.2–18)	5.43 (0.1–16)	.380
Sex			
Male	12	2	.681
Female	5	1	
Lens status			
Phakic	13	2	
Aphakic	3	1	.601
Pseudophakic	1	0	
Characteristics of RRD			
Macula status at diagnosis			
On	10	0	.050
Off	7	3	
Duration			
>3 months	12	0	
>2 months	1	3	.007
<2 months	4	0	
Extent of RD			
Total	5	3	
Subtotal	9	0	.155
Inferior	2	0	
Superior	1	0	
Number of breaks			
Not seen	6	0	
>2	6	3	.089
One	5	0
Type of retinal break			
Tear	11	2	
Hole	3	0	.749
Dialyses	1	0	
Giant tear	2	1	
PVR			
None	6	0	.521
Present	11	3	

## DISCUSSION

Pediatric RRD is much less common than adult retinal detachment. In our institution, a tertiary care ophthalmology facility that serves a catchment population of approximately 5 million, accounted for 9.4% of all retinal detachment surgery cases performed over a period of 3 years. This incidence is higher than reported in studies from other parts of the world. Yokoyama et al^5^ found an incidence of 3.1% of pediatric RRD (age 0-15 years; mean 12 years) of all RRD cases in the Japanese population, while Rumelt et al^6^ found an incidence of 6.6% (age 0-18 years; mean 10.8 years) in a mixed Middle Eastern population of mainly Caucasian descent. This higher incidence in our study may be related to factors such as a higher incidence of inherited myopia/vitreoretinal degeneration in children and ocular trauma in pediatric population due to socioeconomic factors in Saudi Arabia.

Like studies reported from other parts of the world, most pediatric patients in our study were male (14/20, 70%). Blunt trauma was the most notable cause of retinal detachment, responsible for 9/20 (45%) cases. This is higher than reported in other studies^6^ and may be due to a higher incidence of ocular trauma in Saudi Arabia.^8^ Myopia and vitreoretinal degeneration are more common in the Arabian population due to hereditary factors, and this was reflected in our study with 4/20 (20%) cases^9^,^10^ of retinal detachment associated with myopia.

The type of surgical procedure for management of pediatric retinal detachment depends on choice of surgeon and is also dictated by the presence or absence of PVR. We opted to treat most patients with a combination of pars plana vitrectomy and encircling explant, as PVR was present in most patients (14/20, 70%). Since most patients (16/20, 80%) had retinal detachment of more than two months duration and PVR, a combination of PPV and encircling buckle, allowed optimal relief of vitreoretinal traction for successful retinal re-attachment. Statistical analysis of factors associated with anatomical outcome (Table 4) following surgery showed that macular detachment (*P*=.05) and longer duration of retinal detachment (*P*=.007) were associated with worse anatomical outcome. A trend of worse anatomical outcome, though not statistically significant (*P*=.089), was also seen when retinal breaks were not seen or more than two retinal breaks were present. These findings are consistent with other studies^4^,^11^ in that early surgical treatment of retinal detachment is more likely to be successful before significant PVR sets in.

The anatomical and functional results of this study are encouraging. We achieved retinal reattachment in 17/20 (85%) cases, and visual acuity showed significant improvement from preoperative levels (*P*=.018). This is comparable with results reported from other studies. Our study is limited by small numbers and retrospective nature of data collection. However, the results of this study are important for documenting clinical features and management plans in Saudi population, where no previous data exists for such a condition.
